# Magnetic resonance diffusion tensor imaging applied to rat model of contrast-induced acute kidney injury

**DOI:** 10.7717/peerj.10620

**Published:** 2021-02-15

**Authors:** Bin Wang, Junjie Li, Yongfang Wang

**Affiliations:** 1 Department of Medical Imaging, Shanxi Medical University, Taiyuan, Shanxi, China; 2 Department of Medical Imaging, First Hospital of Shanxi Medical University, Taiyuan, Shanxi, China; 3 Department of Radiology, First Hospital of China Medical University, Shenyang, Liaoning, China

**Keywords:** Contrast-induced acute kidney injury, Chronic kidney disease, Diffusion tensor imaging, Hypoxia-inducible factor-1α

## Abstract

**Objectives:**

In this preclinical investigation, the feasibility of using diffusion tensor imaging (DTI) to study contrast-induced acute kidney injury (CIAKI) is explored, comparing radiographic outcomes with histopathologic and immunohistochemical findings after repeated animal exposures to iodinated contrast agent.

**Materials and Methods:**

Forty-five male wistar rats were allocated to three groups (*n* = 15 each), each receiving two separate injections 1 day apart: group 1 (iodixanol then saline); group 2 (iodixanol twice); and control group (saline twice). Five rats were then randomly selected from each group at three separate time points (1 h, 24 h, and 120 h) for magnetic resonance imaging (MRI). Upon MRI completion, the animals were sacrificed, examining renal tissue and serum creatinine level. DTI data served to calculate fractional anisotropy (FA) and apparent diffusion coefficient (ADC).

**Results:**

FA values were significantly lower in group 2 than in the others. Compared with controls, FA assessments at 1 h, 24 h, and 120 h after injections commenced were significantly lower in group 2; and ADC was significantly more pronounced at 24 h. Serum creatinine levels at 24 h were markedly elevated in both groups 1 and 2. Pearson correlation analysis revealed significant negative correlations between FA (*r* = −0.730; *p* < 0.05) or ADC (*r* = −0.827; *p* < 0.05) and tubular injury and between FA (*r* = −0.563; *p* < 0.05) or ADC (*r* = −0.805; *p* < 0.05) and hypoxia-inducible factor-1α.

**Conclusions:**

Analytic approaches to DTI with better reproducibility should aid in monitoring the early pathophysiologic derangements of CIAKI, thus facilitating timely reversal of the detrimental effects.

## Introduction

Nonionic iodinated contrast agents (CAs) are frequently used in medical practice, especially in computed tomography studies and interventional procedures (Zhao et al., 2019a). Although very well tolerated in current realms of routine clinical usage, a variety of side effects may occur after exposures. Contrast-induced acute kidney injury (CIAKI) may develop and has become the third most common cause of hospital-acquired acute renal failure (Van der Molen et al., 2018). CIAKI is associated with significant morbidity and mortality short- and long-term (Fahling et al., 2017) warranting much scientific discussion in recent years and fueling considerable clinical concern.

According to established guidelines, CIAKI is defined as an increase in serum creatinine (SCr), in the absence of overt kidney damage (Chalikias, Drosos & Tziakas, 2016). However, given the inherent delay, SCr is typically assessed 24–72 h after CA delivery (Luders et al., 2015). Earlier reports have shown that diffusion tensor imaging (DTI) is a new technique enabling noninvasive quantitative monitoring of microstructural and functional intrarenal changes. The kidney itself is a particularly amenable to DTI studies, given its primary function of water transport and the anisotropic diffusion properties of its radially oriented vessels, tubules, and collecting ducts. As DTI-derived parameters, fractional anisotropy (FA) and apparent diffusion coefficient (ADC) measure diffusion in at least six different directions, from which the full diffusion tensor and thus the main diffusion direction are calculated. FA and ADC thus provide added information by gauging diffusion direction and degree of directed diffusion (Wang et al., 2018; Li et al., 2015). By determining changes in diffusion, the functional impact of CIAKI may be better assessed. However, DTI has yet to be used in measuring early renal functional consequences of repeated CA dosing.

Renal hypoxia and direct nephrotoxicity of CA are the universally acknowledged causes of CIAKI. Given the well-known fact that tubular vacuolar, tubulointerstitial fibrosis, and tubular damage occur in CIAKI, the hypoxia-inducible transcription factor-1α (HIF-1α) is also highly expressed in CIAKI (Wang et al., 2019). We hypothesize that DTI is able to detect pathological changes of CIAKI and determine the correlation of FA to the degree of renal pathologies and HIF-1α expression. To date, there is no strong or established correlation with two doses of CIAKI and DTI. Furthermore, renal function is not always fully restored from the first injection and the development of long-term renal insufficiency often ensues after AKI. Here, DTI was applied for detection of renal dysfunction in iodixanol-treated rats over a relatively longer duration of time after duplicated injection.

The goal of this pilot imaging study was to explore the feasibility of DTI use for visualizing/quantifying diffusion properties and directions in the setting of CIAKI. A rat model was engaged for this purpose, delivering a single dose of CA to one group of animals and two doses to another, 1 day apart. We also measured expression levels of marker genes signaling renal damage and compared DTI-derived parameters with scored histopathologic changes.

## Materials and Methods

### Animals population

This prospective preclinical study was approved by the China Medical University Ethics Committee (IACUC Issue No. 2018299) and formulated in accordance with the National Institutes of Health Guide for the Care & Use of Laboratory Animals. A total of 45 male wistar rats (weights, 280–320g) were obtained and randomly allocated to three groups (*n* = 15 each) for consecutive injections (1 day apart) as follows: group 1: CA then saline; group 2: CA both days; and control group: saline both days. All iodixanol (Visipaque 320; GE Healthcare, Chicago, IL, USA) injections were intravenous (IV), administered via tail vein at a dosage of 4 g iodine/kg body weight (BW) (Lenhard et al., 2013). The rats were anesthetized via an intraperitoneal injection dose of 3% pentobarbital sodium at 2 mL/kg. The effects of the second application of iodixanol at 1 h, 24 h, and 120 h were evaluated. The control group received equal volumes of normal saline.

### DTI protocol

All rats were fasted for 8 h before MRI examinations. In each group, 5 rats selected at random were subjected to magnetic resonance imaging (MRI) at three separate time points (1 h, 24 h, and 120 h) post injection (PI) (Wang et al., 2017). MRI acquisitions relied on a clinical 3.0T Twin speed scanner (GE Medical Systems, Milwaukee, WI, USA) using an 8-channel wrist coil. All images were performed without respiratory triggering. Respiratory motion and bowel loops were reduced by imaging the rats in the right decubitus position. DTI studies involved single-shot 2D echo planar imaging performed in six directions, with b-values of 0 and 500 s/mm^2^ and respiratory triggering stipulated as follows: repetition time/echo time, 3,175/126 ms; field of view (FOV), 100 × 65 mm^2^; slice thickness, 2.4 mm; matrix, 160 × 160; bandwidth, 125 kHz; number of signal averages (NEX), 4; number of slices, 3; acquisition time, 4 min and 4 s.

**Figure 1:**
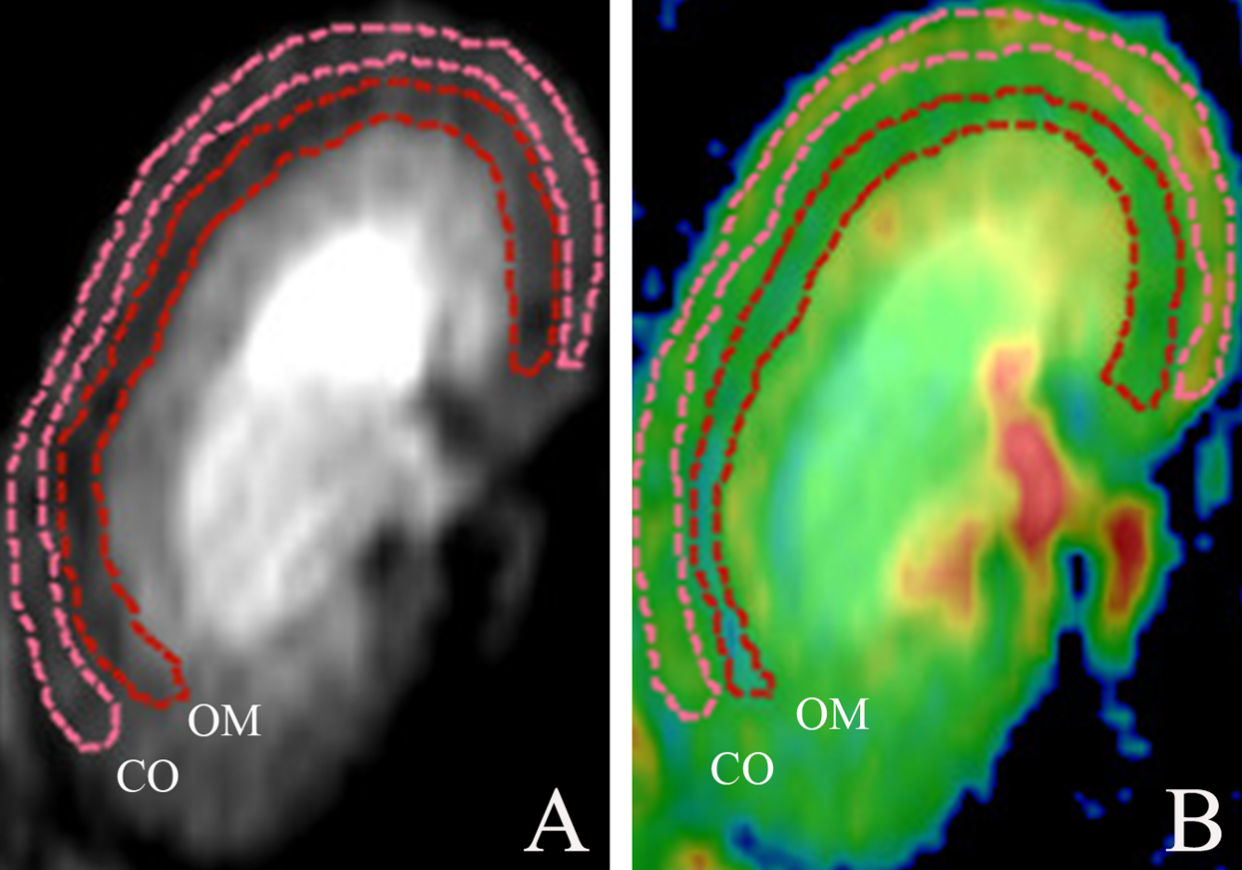
Morphologic MR images with representative cortical and medullary ROI segmentations. (A) ROIs traced on DTI of kidney and (B) ROIs traced on DTI pseudocolor map. DTI, diffusion tensor image; ROI, region of interest; CO, cortex; OM, outer medulla.

Post-processing was achieved using the Functool Software Package (GE Healthcare, Chicago, IL, USA). The diffusion measures along the six axis are fitted to a 3 × 3 symmetric matrix to form a three-dimensional ellipsoid model for DTI analysis. Characteristics of this model are defined by Eigenvectors (v1, v2 and v3) and Eigenvalues (1, 2 and 3), so as that analysis of the diffusion along the main axis can be conducted (Hueper et al., 2012). The extent of diffusion anisotropy is calculated and depicted in the FA parameter maps, and it is graded from 0 (no favorable diffusion orientation, isotropic diffusion) to 1 (only one diffusion direction, complete anisotropic diffusion). Based on a monoexponential fitting model, the apparent diffusion coefficient maps are calculated. Quantitative measurements of FA and ADC values were generated by an experienced abdominal radiologist responsible for (and blinded to) all data collection. Regions of interest (ROIs) were manually traced for anatomic layers of renal cortex (CO) and outer medulla (OM), drawn separately along organ contours (Fig. 1) (Liang et al., 2016), and mapped in triplicate (each nearly identical in shape and size (18–22 mm^2^)) on DTI images (Wang et al., 2017). Mean FA and ADC values in each animal were separately determined.

### Biochemical assay

All rats were sacrificed at specified time points after MRI examinations. Blood was extracted from inferior vena cava (0.5–1.0 mL) standing in room temperature (20–25 °C) for 30 min and centrifuged at 3,500 rmp for 10 min, collecting serum for creatinine analysis serum creatinine concentration at a local clinical laboratory using an automatic biochemical analyzer according to the manufacturer’s instructions.

### Histopathologic examination

Sections of right kidney were subjected to routine tissue processing, then embedded in paraffin and sectioned (5 μm) for hematoxylin and eosin (H&E) staining. The slides prepared were examined in blinded manner, selecting five fields of renal cortex and medulla (200× magnification) at random to gauge the extent of morphologic changes. Renal injury (i.e., tubular vacuolar change, tubular necrosis, tubular casts, inflammatory cells, renal interstitial fibrosis, and tubular dilatation) was scored on a scale of 0–4 as follows: 0 (normal kidney); 1 (<5% of renal injury); 2 (5–25% of renal injury); 3 (25–75% of renal injury); and 4 (>75% of renal injury) (Ulusoy et al., 2014).

### Immunohistochemical staining

Formalin-fixed, paraffin-embedded sections of kidney were used for immunostaining of hypoxia-inducible factor-1α (HIF-1α). After 20-min Tris-EDTA (pH 9.0) incubation at 95 °C for antigen retrieval, the slides were then blocked in 2% normal horse serum for 30 min, followed by 20 min in 3% hydrogen peroxide. Primary antibody HIF-1α (NB100-105, 1:100 dilution; Novus Biologicals, Littleton, CO, USA) was applied at room temperature for 120 min. Biotin-labeled goat anti-mouse IgG served as secondary antibody, using diaminobenzidine (DAB) as chromogenic substrate. The brownish precipitates at points of peroxidase localization were interpreted using the smart segmentation tool of ImageJ (National Institutes of Health, Bethesda, MD, USA) open-source software (Wang et al., 2019).

### Statistical analysis

Data collected were expressed as mean ± standard deviation values. All computations were driven by standard software (SPSS v22.0; IBM, Armonk, NY, USA), setting significance at *p* < 0.05; and all data were tested for normality. In group-wise assessments of FA or ADC, one-way analysis of variance (ANOVA) was used, conducting *post-hoc* comparisons via Fisher’s Least Significant Difference (normal distributions) or Kruskal–Wallis test (non-normal distributions). Pearson’s analysis was applied to examine correlations between DTI-derived variables (FA, ADC) and histopathologic changes.

## Results

### Early serum creatinine variations

Serum creatinine determinations are listed in Table 1. Significant differences in SCr levels were demonstrated by the three groups over time, peaking at 24 h. Mean SCr levels at 24 h in groups 1 and 2 were 35.58 ± 4.04 µmol/L and 42.56 ± 4.67 µmol/L, respectively, both somewhat or significantly higher than the control group mean (30.32 ± 3.96 µmol/L; *p* = 0.095 and *p* = 0.001, respectively). Group 2 significantly surpassed group 1 (*p* = 0.033) and the control group (*p* = 0.001) in this regard. At 120 h, mean SCr levels of groups 1 and 2 were 31.64 ± 2.09 µmol/L, and 36.74 ± 2.97 µmol/L, respectively, group 2 again significantly surpassing group 1 (*p* = 0.032) and the control group (29.97 ± 4.06µmol/L; *p* = 0.007).

**Table 1:** Serial serum creatinine (µmol/L) determinations by group.

	1h		24h		120h	ANOVA, *P*
Group 1	30.86 ± 1.51		35.58 ± 4.04		31.64 ± 2.09	
Group 2	33.46 ± 3.64	0.107,0.254	42.56 ± 4.67*^,^^#^	0.001,0.033	36.74 ± 2.97*^,^^#^	0.007,0.032
Control group	29.18 ± 4.65		30.32 ± 3.96		29.97 ± 4.06	

**Notes:**

* *p* < 0.05 vs group 1.

# *p* < 0.05 vs control group.

### Functional DTI analysis

FA and ADC values of group 1, group 2 and the control group are provided in Table 2. In all animals, the two anatomic layers of kidney (CO and OM) were identifiable via DTI.

**Table 2:** Serial determinations of DTI-derived parameters by group.

Parameters	Renal regions	Treatment	1 h	24 h	120 h
		Group 1	0.284 ± 0.0213	0.290 ± 0.00822	0.294 ± 0.0359
	CO	Group 2	0.236 ± 0.0157*^,^^#^ (0.000,0.003)	0.259 ± 0.0176*^,^^#^ (0.000,0.002)	0.292 ± 0.00467 (0.471,0.937)
		Control group	0.307 ± 0.0229	0.303 ± 0.0100	0.305 ± 0.0225
FA		Group 1	0.433 ± 0.0142^#^ (0.000)	0.522 ± 0.0200^#^ (0.000)	0.571 ± 0.00259^#^ (0.000)
	OM	Group 2	0.394 ± 0.00476*^,^^#^ (0.000,0.000)	0.483 ± 0.00422*^,^^#^ (0.000,0.000)	0.510±0.0342* (0.000, 0.084)
		Control group	0.588 ± 0.0119	0.598 ± 0.00907	0.595 ± 0.00638
		Group 1	2.218 ± 0.102^#^ (0.025)	2.370 ± 0.253 (0.214)	2.412 ± 0.104 (0.973)
	CO	Group 2	1.968 ± 0.156*^,^^#^ (0.000,0.000)	2.222 ± 0.132 (0.065,0.366)	2.403 ± 0.232 (0.865,0.937)
ADC		Control group	2.462 ± 0.181	2.542 ± 0.323	2.422 ± 0.147
(×10^-3^mm^2^/s)		Group 1	2.094 ± 0.230 (0.071)	2.288 ± 0.108 (0.214)	2.390 ± 0.280 (0.973)
	OM	Group 2	1.780 ± 0.181*^,^^#^ (0.001, 0.046)	2.096 ± 0.184^#^ (0.064, 0.006)	2.288 ± 0.145 (0.525, 0.504)
		Control group	2.374 ± 0.254	2.412 ± 0.145	2.385 ± 0.255

**Notes:**

DTI, diffusion tensor imaging; FA, fractional anisotropy; ADC, apparent diffusion coefficient; CO, cortex; OM, outer medulla.

* *p* < 0.05 vs group 1.

# *p* < 0.05 vs control group.

### Fractional anisotropy profiles

**Figure 2:**
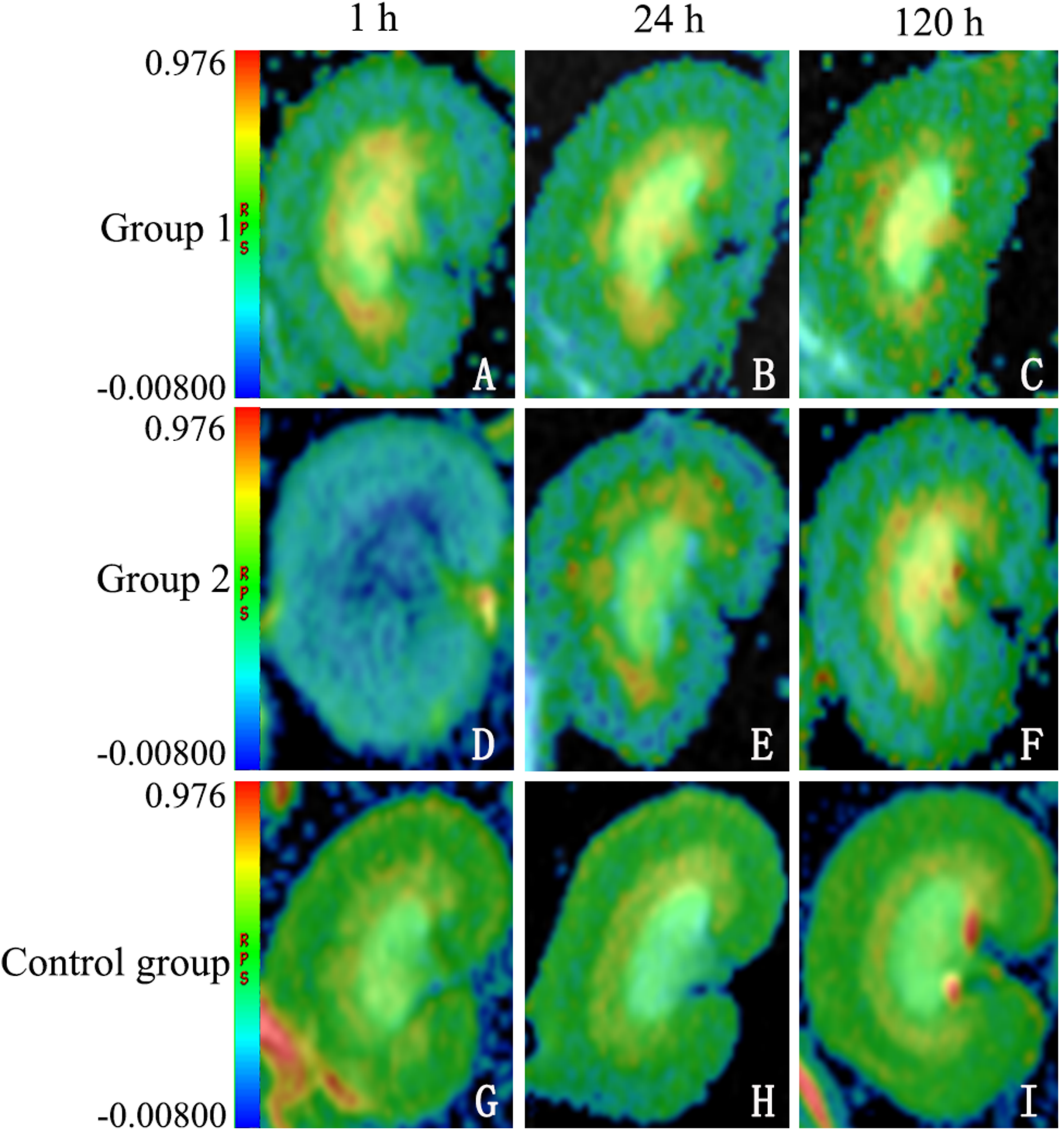
Sample diffusion maps of animal groups, shown chronologically. The lower fractional anisotropy (FA) values of DTI maps indicating higher diffusion anisotropy in group 2 (D–F), compared with group 1 (A–C) and control group (G–I) (note: all maps displayed in same window). DTI, diffusion tensor imaging.

Overall, FA determinations proved significantly lower in group 2 than in the other groups. At 1 h, FA values were significantly lower in both test groups, whether at CO (group 1: 0.284 ± 0.0738 (*p* = 0.09); group 2: 0.236 ± 0.0140 (*p* < 0.0001)) or at OM (group 1: 0.433 ± 0.115 (*p* < 0.0001); group 2: 0.394 ± 0.00426 (*p* < 0.0001)), compared with controls (CO: 0.307 ± 0.0651; OM: 0.588 ± 0.135). FA values in group 2 also significantly exceeded those in group 1, whether at CO (*p* = 0.003) or at OM (*p* < 0.0001). At 24 h, FA determinations again were significantly lower in both test groups at CO (group 1: 0.290 ± 0.0743 (*p* = 0.141); group 2: 0.259 ± 0.0158 (*p* < 0.0001)) and at OM (group 1: 0.522 ± 0.140 (*p* < 0.0001); group 2: 0.483 ± 0.00377 (*p* < 0.0001)), compared with controls (CO: 0.303 ± 0.0100; OM: 0.598 ± 0.00907); and they were significantly greater in group 2 than in group 1 at CO (*p* = 0.002) and at OM (*p* < 0.0001). At 120 h, FA values once more were significantly lower in both test groups at CO (group 1: 0.294 ± 0.0359 (*p* = 1.000); group 2: 0.259 ± 0.0158 (*p* = 0.558)) and at OM (group 1: 0.571 ± 0.00259 (*p* < 0.0001); group 2: 0.510 ± 0.0342 (*p* < 0.0001)), compared with controls (CO: 0.305 ± 0.0225; OM: 0.595 ± 0.00638). However, only FA values at OM differed significantly in the test groups, group 2 surpassing group 1 (*p* < 0.0001) (Fig. 2).

### Apparent diffusion coefficient profiles

Renal ADC values (×10^−3^mm^2^/s) also showed similar trends in all groups (Table 2). At 1 h, ADC values were significantly lower in both test groups at CO (group 1: 2.218 ± 0.102 (*p* < 0.0001); group 2: 1.968 ± 0.156 (*p* = 0.025)) and at OM (group 1: 2.094 ± 0.230 (*p* = 0.0001); group 2: 1.780 ± 0.181 (*p* = 0.071)), compared with controls (CO: 2.462 ± 0.181; OM: 2.374 ± 0.254); and ADC values in group 2 were higher than those in group 1 at CO (*p* = 0.022) or at OM (*p* = 0.046). At 24 h, the ADC values at OM was significantly lower in group 2 (2.096 ± 0.184) than in controls (2.412 ± 0.145; *p* = 0.006). At 120 h, ADC values declined slightly at CO and at OM in all three groups, but observed differences did not reach statistical significance.

### Histopathologic outcomes in three animal groups

#### Renal tissue manifestations

H&E-stained sections of tissue exposed to iodixanol showed substantial vacuolar degeneration of tubules at CO, with tubular casts, detachment of tubular cells, renal interstitial fibrosis, and intraluminal desquamation at OM. Tubular necrosis was severe in iodixanol-retreated rats of group 2, conferring high tubular injury scores.

**Figure 3:**
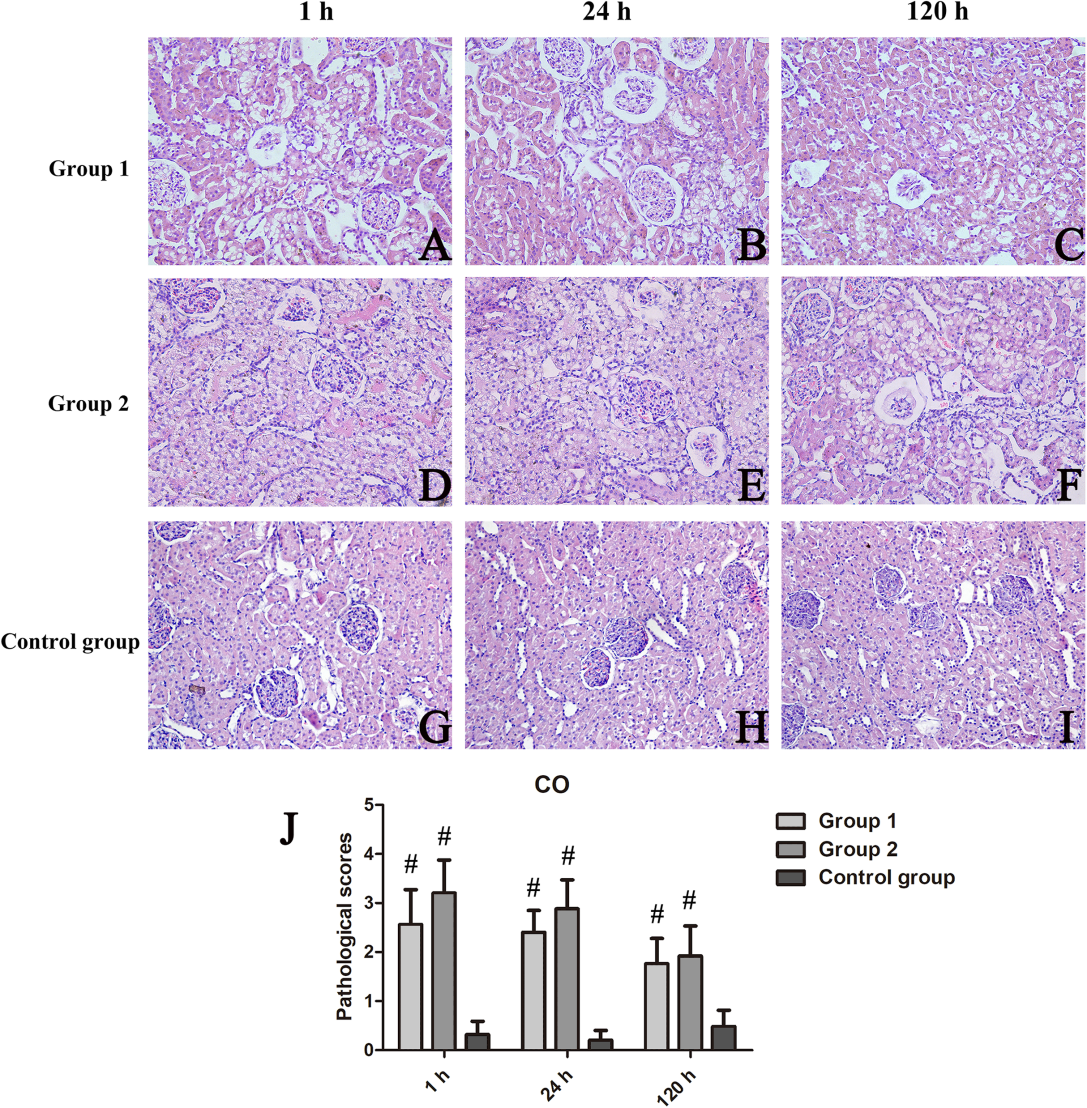
Histologic features of renal cortex over time, shown by animal group (200×). (A–I) H&E-stained tissue sections revealing vacuolar change of renal tubules in contrast-treated rats, with inflammatory cell influx, cellular casts, and glomerular atrophy; and (J) Cortical damage scores by group. ^#^*p* < 0.05 vs control group.

**Figure 4:**
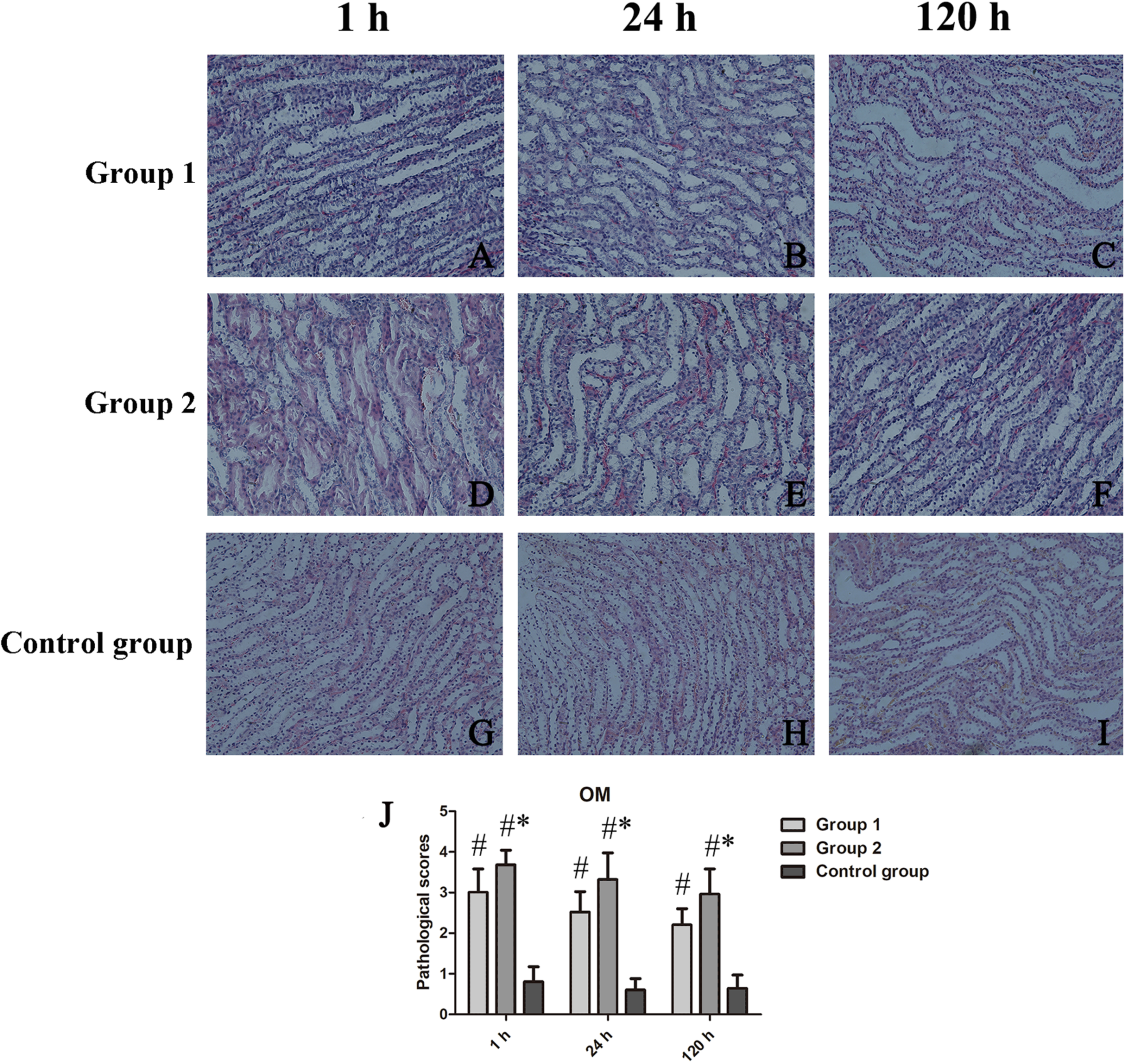
Histologic features of renal medulla over time, shown by animal group (200×). (A–I) H&E-stained tissue sections showing tubular necrosis, tubular vacuolization, tubulointerstitial fibrosis, and proteinaceous casts in contrast-treated rats; and (J) Medullary damage scores by group. *p < 0.05 vs group 1; ^#^*p* < 0.05 vs control group.

At CO, kidney injury scores of groups 1 and 2 and those of the control group differed significantly (*p* < 0.001 each). Scored renal histopathology at 1 h in group 2 surpassed the 1 h scores in group 1 but failed to show significance (*p* = 0.111). At 24 h, significant renal injury (*p* = 0.001) was evident in group 2, compared with controls, whereas groups 1 and 2 did not differ significantly (*p* = 0.113); The same was true at 120 h, significant tubular vacuolar degeneration (*p* = 0.000) appearing in group 2 (vs controls), groups 1 and 2 showing similarities (*p* = 0.623) (Fig. 3). Within OM, kidney injury scores were worse and tubular injury was more serious than within CO. Compared with controls, kidney injury scores of groups 1 and 2 were significantly higher at 1 h (*p* = 0.035), 24 h (*p* = 0.028), and 120 h (*p* = 0.025) (Fig. 4).

#### Scored HIF-1α activity

**Figure 5:**
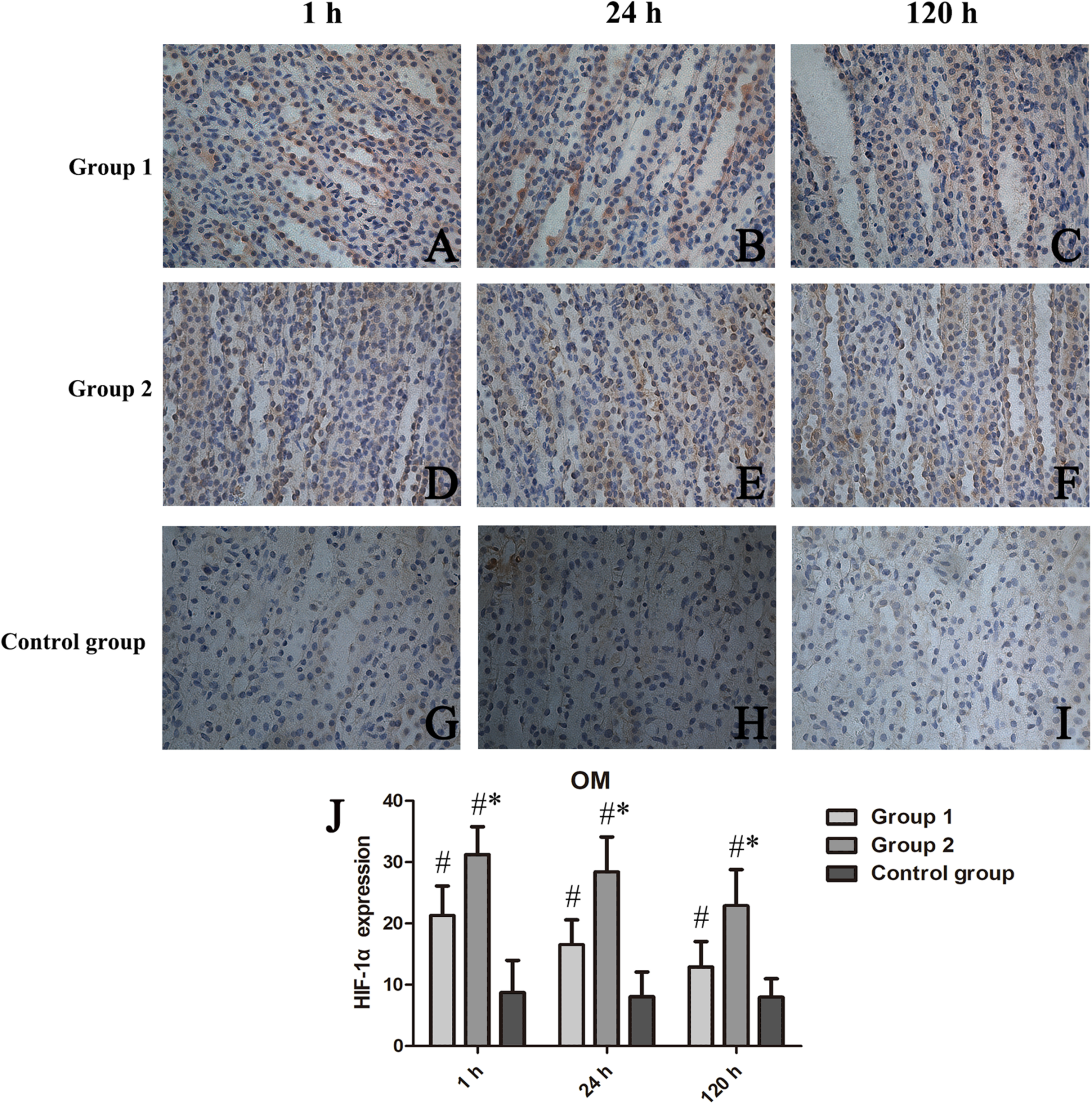
Chronologic views of renal medullary HIF-1α immunostaining in three animal groups (400×). (A–I) HIF-1α expressed primarily in group 2 surpassing others; and (J) Relative quantitation shown as percentage of cells displaying HIF-1α positivity in high-power field (Image J). HIF-1α, hypoxia-inducible factor-1α; *p < 0.05 vs group 1; ^#^*p* < 0.05 vs control group.

HIF-1α expression was confined to OM, as was our analysis of scored expression levels presented in Fig. 5. Similar to the functional and histologic changes of kidney already described, rats given two iodixanol injections displayed significant increases in renal HIF-1α activity, compared with control animals or those receiving single iodixanol injections. Mean numbers of renal tubular cells positive for HIF-1α were significantly greater in group 2 (vs group 1) at 1 h, 24 h, and 120 h (*p* = 0.008, *p* = 0.002, and *p* = 0.005, respectively).

#### Correlating DTI parameters with renal histopathology and HIF-1α scores

**Figure 6:**
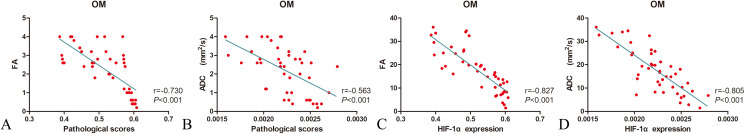
Correlating FA and ADC values with histopathologic scores and HIF-1α expression levels in three animal groups. (A) FA and histopathologic scores; (B) ADC and histopathologic scores; (C) FA and HIF-1α expression; and (D) ADC and HIF-1α expression FA, fractional anisotropy; ADC, apparent diffusion coefficient; HIF-1α, hypoxia-inducible factor-1α.

As illustrated in Fig. 6, tubular injury (scored during tissue assessments) correlated significantly with FA values at OM (*r* = −0.730; *p* < 0.001) in all animals, as did HIF-1α expression levels and FA values at OM (*r* = −0.827; *p* < 0.001). In all animals, tubular injury and ADC values at OM correlated moderately (*r* = −0.563; *p* < 0.001), whereas a strong positive correlation was shown between HIF-1α expression levels and ADC values at OM (*r* = −0.805; *p* < 0.001).

## Discussion

MR diffusion imaging is a sensitive tool for characterizing the random microscopic motion of water molecules and has now prevailed in routine MRI protocols applied to human and animal brain studies (Tsai et al., 2019; Liu et al., 2020; Zhang & Liu, 2020). The concept of using MR diffusion imaging to characterize the kidney is intriguing and potentially important, considering that water transport is critical for kidney function and shows substantial architectural directionality (Notohamiprodjo et al., 2008). Outcomes of the present study, utilizing MR DTI in a rat model of CIAKI, seem to confirm that tubular damage negatively correlates with diffusion anisotropy. At acute stages of CIAKI, reliable estimates of regional renal injury are thus achievable by DTI.

Our data indicate greater depression of FA in test groups 1 and 2, compared with control animals, although the pathologic mechanisms implicated in such shifts are uncertain (Wang et al., 2017). Still, it doses appear that declines in renal perfusion are in part to blame for FA reductions after repeated iodixanol exposure and pertain to all renal structures (Chen et al., 2015; Wu et al., 2017). We suspect that imbalances of this sort dictates the extent of renal hypoxia and the proclivity for acute renal disease (Lenhard et al., 2013; Calvin, Misra & Pflueger, 2010). Such perfusion deficits, fueled by impaired microcirculatory convective oxygen transport and erythrocytic are likely instigators of renal hypoxia (Persson, Hansell & Liss, 2005). A recent report linking repeated CA use to renal hypoperfusion in pigs offers some corroboration (Haneder et al., 2012).

Others have speculated that tubular casts and cellular debris seen in acutely injured animal kidneys hinder the directionality of tubular diffusivity and thus contribute to FA restriction (Cheung et al., 2010). A strong correlation with between FA and renal injury scores of OM (*r* = −0.730) has indeed emerged in the present study; and as our tissue sections (H&E) attest, parenchymal damage caused by interstitial fibrosis may also alter diffusivity and impair renal function. A relation between faltering and tissue fibrosis has actually been demonstrated in a prior rat model of diabetic nephropathy (Hueper et al., 2012). According to another study by Rollins et al. (2017) declines in FA likely stem from necrosis/apoptosis of medullary tubular epithelial cells, impairing water diffusion along tubular structures. Cheung et al. (2010) have further acknowledged reduced renal medullary FA values 5 h after ischemia-reperfusion injury, accompanied by cellular edema and histologic evidence of necrosis. Although inflammatory cells abounded in our tissue samples of group 2 test animals, the reason for this influx in the context of CIAKI is perplexing. Surges in complement activation may be responsible and should be addressed in the future.

Our study data clearly demonstrate the aftereffects of iodixanol exposure on renal FA and ADC values in both animal test groups. The most profound injurious changes, encountered within OM, are thought to precipitate acute renal failure. It is possible that iodixanol promotes renal intrarenal vasoconstriction and hypoperfusion, thereby restricting the diffusion of water molecules. HIF-1α is highly expressed in OM, suggesting marked hypoxia in response to FA and ADC reductions. Thus, the observed DTI data correlated well with both histologic changes and HIF-1α expression in renal tissues. This has important implications for human disease. Any pharmacologic agent predisposing to medullary injury may risk future susceptibility to acute renal damage.

At 120 h after repeat administration, we evaluated the impact of CA on the dynamics of renal water diffusion and direction. Throughout the investigation, there were steep OM declines in renal FA values of group 2, whereas corresponding FA values were consistently lower in group 1 by comparison. These outcomes imply that repetitive injection of iodixanol within short time windows may induce acute kidney injury (AKI) and impose long-term adverse results. However, the reason that multiple episodes of AKI accelerate long-term kidney injury is obscure. First, renal elimination of iodine was delayed after administering iodixanol, chiefly due to viscosity (its main physicochemical property) (Seeliger et al., 2007), which likely impairs the tubular system and causes FA values to decline. Second, it is also generally acknowledged that such agents disrupt the oxygen balance of renal medulla, triggering hypoxic damage in predisposed patients (Persson, Hansell & Liss, 2005). A specific histopathologic feature of AKI is significant overexpression of HIF-1α by tubular epithelial cells, signaling CA toxicity and medullary hypoxia (Loeffler & Wolf, 2015). Such injury to medulla is thought to precipitate acute renal failure. In animal models, HIF-1α expression level seems to reflect acute renal injury-to-chronic kidney disease (AKI-to-CKD) transition. As one of the most upregulated proteins in the kidney, HIF-1α may be a promising biomarker for predicting risk of CKD after AKI.

Incomplete clinical recovery from AKI may promote a long-term decline in kidney function. The biochemical pathways related to such progression have attracted a great deal of attention (Maekawa & Inagi, 2019). According to Maioli et al. (2012) occurrences of CIAKI seemingly predispose patients to repeated, sustained episodes of kidney injury, with high risk of rehospitalization. Furthermore, there is some documentation that prolonged (7-year) elevations of IL-18, kidney injury molecule-1, and liver-type fatty acid-binding protein may be harbingers of AKI in children (Cooper et al., 2016). The molecular underpinnings of AKI-to-CKD transition are as follows: (1) mitochondrial injury and metabolic disorders inflicted by AKI may foster maladaptive repair in the aftermath (Zhao et al., 2019b); (2) persistent inflammation at early stages inevitably results in progression to CKD (Maekawa & Inagi, 2019); (3) fibroblast activation and deposition of extracellular matrix as triggers of renal fibrosis eventuate in CKD (Coca, Singanamala & Parikh, 2012); and (4) endothelial dysfunction, vasoconstriction, and vascular congestion may incite a vicious cycle, undermining the microvascular circulation (Basile, Collett & Yoder, 2018).

Although iodixanol was injected at a dose of 4 g iodine/kg body weight, just as in previous study (Lenhard et al., 2013). This dosage yields an equivalent dose of human exposure during CA-enhanced CT (standard range: 0.5–0.8 g iodine/kg) due to the applied dose was incorporated FDA Toxicology Guidelines that requiring bodily surface area normalization (rat:human = 6:1) (Wang et al., 2014). Our study still has certain limitations. First, the sample size was relatively small, with only 5 rats per group sacrificed at each juncture. However, through detailed longitudinal and cross-sectional examination, temporal changes within differing zones of the kidneys were well demonstrated by DTI after CA exposures, correlating with histopathologic changes and HIF-1α expression. Another issue is that CO and OM ROIs were delineated based on FA maps and our knowledge of renal anatomy, rather than using high-contrast anatomic images. Finally, the complex mechanisms involved in development of CIAKI make it difficult to determine FA values or shifts in ADC with precision. Nevertheless, we are convinced that variable degrees of glomerular or tubular injury and tubulointerstitial fibrosis may alter the renal microstructure over time, and that FA and ADC determinations are useful for assessing the cumulative effects of such changes.

Forthcoming technologic innovations, such as arterial spin labeling, intravoxel incoherent motion, and blood-oxygen-level-dependent imaging, are awaited to clarify how changes in renal microstructure and function influence DTI parameters (especially FA) and should enable acquisition of true DTI-based metrics. Unlike the intuitively gauged diffusion properties recorded in our animal groups, these may be critical when investigating potential renal injury. Partial volume effect also limited our ability to optimize DTI parameters and should be a focus of research going forward.

In conclusion, we found DTI useful for assessing temporal changes of CIAKI in various animal groups. As a DTI-derived parameter, FA helped with early detection and monitoring of renal damage after repeated CA injection of rats. Ultimately, our understanding of functional recovery from AKI is pivotal in preventing AKI-to-CKD transition, unleashing substantial therapeutic and economic benefits.

## Supplemental Information

10.7717/peerj.10620/supp-1 Supplemental Information 1 The raw data of DTI-derived parameters in three groups. FA: fractional anisotropy; CO: cortex; OM: outer medulla.

10.7717/peerj.10620/supp-2 Supplemental Information 2 The raw data of DTI-derived parameters in three groups. ADC: apparent diffusion coefficient; CO: cortex; OM: outer medulla.

## Additional Information and Declarations

### Data Availability

The following information was supplied regarding data availability: Raw data are available in the Supplemental Files.
